# Complete mitochondrial genome analysis of *Distolasterias nipon* (Echinodermata, Asteroidea, Forcipulatida)

**DOI:** 10.1080/23802359.2018.1501313

**Published:** 2018-10-25

**Authors:** Taekjun Lee, Sook Shin

**Affiliations:** a Marine Biological Resource Institute, Sahmyook University, Seoul, Korea;; b Division of Life Sciences, College of Life Sciences and Biotechnology, Korea University Korea University, Seoul, Korea;; c Department of Chemistry Life Science, Sahmyook University, Seoul, Korea

**Keywords:** Echinodermata, Asteriidae, Sea star, Complete mitogenome

## Abstract

In this study, we determined the complete mitochondrial genome sequences of *Distolasterias nipon* belonging to the class Asteroidea in the phylum Echinodermata. The complete mitogenome of *D. nipon* was 16,193 bp in length and consisted of 13 protein-coding genes (PCGs), 22 tRNAs, and 2 rRNAs. The orders of PCGs and rRNAs were identical to those of the eight recorded mitogenomes of asteroids. The phylogenetic tree determined by the maximum likelihood method revealed that *D. nipon* was clearly grouped with *Aphelasterias japonica* and *Asterias amurensis*, which belong to the family Asteriidae.

The class Asteroidea, belonging to phylum Echinodermata, is composed of approximately 1,500 species from polar waters to tropical waters (Clark and Downey 1992; Clark and Mah 2001). The genus *Distolasterias* contains four species worldwide and distributed in the North Pacific. These species include eurybathic sea stars from the shore to the deep sea to under 900 m (Fisher 1911; D’yakonov 1968; Shin 2010). The mitochondrial complete genomes (mitogenome) of asteroid species have been reported for only eight species to date (Asakawa et al. 1995; Matsubara et al. 2005; Yasuda et al. 2006; Seixas et al. 2018). 

Specimens were collected by technical diving to a depth of 54 m in the East Sea of Korea (37°50′55″N, 128°56′26″E). The specimens were deposited in the Marine Echinoderm Resources Bank of Korea (Seoul, Korea). The mitochondrial DNA was extracted using the Qproteome^®^ Mitochondria Isolation kit (Qiagen, Hilden, Germany) according to the manufacturer’s instructions and isolated using a QIAamp DNA mini kit (Qiagen). Mitochondrial DNA was amplified using REPLI-g Mitochondrial DNA Kit (Qiagen). Next-generation sequencing (NGS) analysis was performed by genome analysis units at the National Instrumentation Centre for Environmental Management of Seoul National University in Korea. A genomic library was constructed from the genomic DNA using a Kapa Hyper Prep Kit (Kapa Biosystems, Woburn, MA, USA) with paired end reading, followed by NGS on the Illumina Hi-Seq 2500 platform (San Diego, CA, USA). Phylogenetic analysis for the dataset was performed using maximum likelihood (ML) with PhyML 3.1 (Guindon et al. 2010). The best-fit substitution was estimated using jModelTest 2.1.1 (Guindon and Gascuel 2003; Darriba et al. 2012) for the nucleotide dataset of 13 protein-coding genes (PCGs). For ML analyses, PhyML was used with the GTR + I + G model of substitution for the nucleotide dataset. Bootstrap resampling was carried out using the rapid option with 1,000 iterations.

The complete mitogenome of *D. nipon* (GenBank accession No. MG464589) was 16,193 bp in length and contained 13 PCGs, 22 tRNA genes, and two rRNA genes. The order and direction of the genes were identical to those of the other eights complete mitogenomes of asteroid species. Twelve PCGs contained an ATG initiation codon (Methionine), with the exception of NADH4L which was initiated with an ATT codon (Isoleucine). The termination codon of the 11 PCGs was TAA codon, with the exceptions of NADH6 (TAG) and CytB (Phenylalanine (TTT) + T).

To examine phylogenetic relationships, we performed the ML method based on the nucleotide sequences of the 13 PCGs obtained from nine asteroids including *D. nipon* (Figure 1). Two ophiuroids, *Astrospartus mediterraneus* (NC_013878) and *Ophiura luetkenii* (NC_005930), were used outgroups. In the phylogenetic tree, *D. nipon* was distinctly grouped with *Aphelasterias japonica* (NC_025766) and established a monophyletic clade with *Asterias amurensis* (NC_006655), which belongs to same family, Asteriidae (Figure 1).

**Figure 1.**
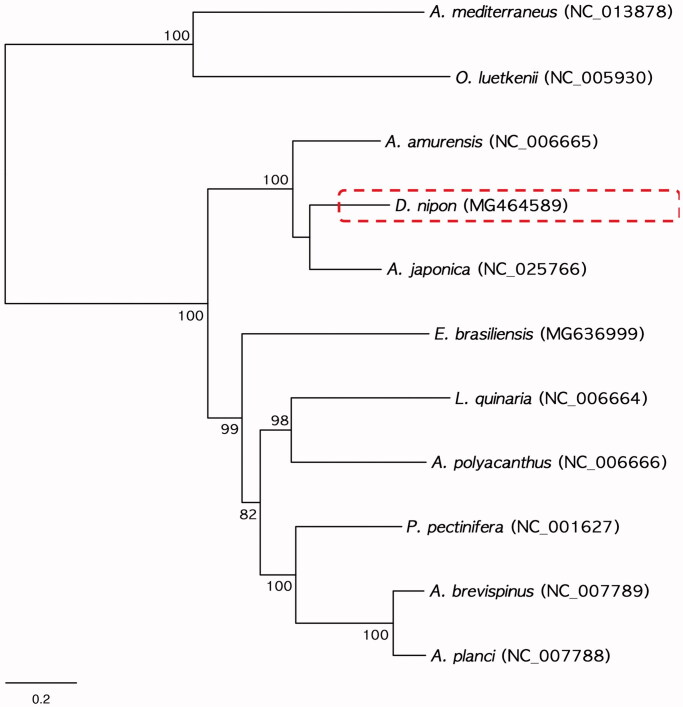
Phylogenetic tree of maximum likelihood (ML) method based on the nucleotide sequences of the complete mitogenomes of *D. nipon* (MG464589) and eight other asteroids. The values of bootstrap support (ML) is indicated on each node as >70.
